# ID 206 - Monitoramento do Horizonte Tecnológico (MHT) de Inibidores de Janus Quinase (JAKi) para o Tratamento de Mielofibrose

**DOI:** 10.5327/2237-9622.2025.v34s1.206

**Published:** 2025-11-25

**Authors:** Renato Picoli, Renato Mantelli Picoli, Renata Ferreira, Júlia Lima, Pedro Antonio Barbosa, Aline Rocha, Natália Brandão, Luiza Costa, Hellen Miyamoto

**Keywords:** monitoramento do horizonte tecnológico, inibidores de Janus quinase, mielofibrose

## Abstract

**Introdução:**

A mielofibrose é uma neoplasia mieloproliferativa caracterizada pela disfunção da medula óssea devido à formação e ao acúmulo de tecido fibroso. Entre as causas estão mutações nos genes JAK2, que levam a uma ativação excessiva da via de sinalização JAK-STAT, resultando na proliferação anormal de células. Isso faz dos inibidores de JAKi um alvo terapêutico relevante para o tratamento da doença. Atualmente, apenas ruxolitinibe está aprovado pela Agência Nacional de Vigilância Sanitária (Anvisa) para o tratamento da mielofibrose. Este MHT visa identificar, de forma sistemática, os JAKi em desenvolvimento para tratar a condição.

**Método:**

As bases de dados ClinicalTrials.gov, WHO e Cochrane foram utilizadas para o levantamento de os registros de ensaios clínicos. Foram utilizadas estratégias de busca com vocabulário controlado e seus respectivos sinônimos relacionados aos JAKi e à mielofibrose. Não foram aplicadas restrições do tipo temporal, idioma ou status do estudo. Foram incluídos os ensaios clínicos de fase 2 e 3 e excluídos os estudos observacionais.

**Resultados:**

Cinco JAKi estão em desenvolvimento para o tratamento da mielofibrose. Lestaurtinibe, que inibe JAK2 e TK3, foi avaliado no ensaio clínico de fase 2 (NCT00494585), já concluído com resultados. Jaktinibe, inibidor de JAK1, JAK2 e JAK3, foi analisado no ensaio de fase 2 (NCT04217993), também concluído com resultados, e está sendo avaliado em um ensaio de fase 3 (ChiCTR2100046946), que ainda está recrutando. Itacitinibe, um inibidor seletivo de JAK1, foi avaliado no ensaio de fase 2 (NCT03144687), concluído com resultados. NS-018, inibidor de JAK2, é indicado para mielofibrose primária, pós-policitemia vera ou pós-trombocitemia essencial, e foi estudado em um ensaio de fase 1/2, já concluído com resultados. Por fim, TQ05105, inibidor de JAK2, é indicado para mielofibrose de risco moderado a grave e está sendo investigado em três estudos: NCT06245941 de fase 1/2 (ainda não recrutando), NCT06122831 de fase 1/2 (já recrutando) e NCT05020652 de fase 2 (com status desconhecido).

**Conclusão:**

Pelo fato de a via JAK-STAT estar ativa de modo aumentado na condição, esses inibidores representam um potencial tratamento para os pacientes com mielofribose. Lestauritnibe apresentou taxa elevada de eventos adversos graves, e o estudo foi de braço único. O estudo de jaktinibe também foi de braço único, possui tamanho amostral e tempo de acompanhamento insuficientes, apesar dos resultados promissores. Itacitinibe, NS-018 e TQ05105 continuam sendo avaliados em seus respectivos ensaios. Dentro do panorama de tratamento da mielofibrose, os JAKi são considerados como medicamentos modificadores da doença promissores. No entanto, os estudos clínicos ainda precisam avançar para estabelecer evidências claras sobre os reais benefícios clínicos dos iJAK para pacientes em condições de doença intermediária e avançada.

